# How do pharmacists use and recommend vitamins, minerals, herbals and other dietary supplements?

**DOI:** 10.1186/s12906-019-2637-y

**Published:** 2019-08-22

**Authors:** Srujitha Marupuru, David Rhys Axon, Marion K. Slack

**Affiliations:** University of Arizona College of Pharmacy, 1295 N Martin Ave, PO Box 210202, Tucson, AZ 85721 USA

**Keywords:** Complementary and alternative medicine, Pharmacists, Vitamins & minerals, Herbals

## Abstract

**Background:**

Use of complementary and alternative medicine (CAM), including vitamins, minerals, herbals, and other dietary supplements, is widespread in the United States (ranging from 24% in Hispanics to 50% in American Indians). Pharmacists are an accessible source for healthcare information, but little is known about their use of CAM products and to whom they would recommend these products.

**Methods:**

A cross-sectional survey was sent via email to pharmacists licensed in one state in the United States in 2015. The survey included items about their use of 10 vitamins and minerals, and 21 herbal or other dietary supplements, as well as reasons for use, conditions used to treat, if they would recommend the product to patients, family, or friends, their perception of CAM safety and effectiveness, and four demographic questions. Descriptive statistics were used to summarize the data, and a chi-square test was used to determine differences between pharmacists’ use of vitamins/minerals and herbals/other dietary supplements. The a priori alpha level was 0.05.

**Results:**

A total of 639 pharmacists completed the survey. Female pharmacists used vitamins/minerals (*p* = 0.031) and herbals/others (*p* = 0.039) more than male pharmacists. Older pharmacists used herbals/others more than younger pharmacists (*p* < 0.001). Fifty-nine percent thought the dietary supplements in the survey were safe while 32% reported they were effective. Seventy-eight percent of respondents reported use of any vitamin or mineral product versus 42% who reported use of any herbal or other dietary supplement. Commonly used products included: multivitamins (91%), vitamin C (71%), fish oil (65%), probiotics (53%), and fiber (53%). The most commonly reported reason for use was general health and wellness (17–90%). Pharmacists most commonly recommend fiber/psyllium (94%) and calcium (90%) to patients, family, and friends.

**Conclusions:**

Pharmacists in this survey selectively used vitamins, minerals, herbals and other dietary supplements, and recommended some of the more commonly used products to patients, family and friends. This is valuable information given that pharmacists are frontline healthcare professionals who may be asked to provide advice about these products.

**Electronic supplementary material:**

The online version of this article (10.1186/s12906-019-2637-y) contains supplementary material, which is available to authorized users.

## Background

The National Institutes of Health (NIH) has defined complementary and alternative medicine (CAM) as “a group of diverse medical and health care systems, practices, and products that are not presently considered to be part of conventional medicine” [1, 2]. Dietary supplements, which include vitamins, minerals, and herbals among others, are a common type of CAM. [3] It is difficult to obtain reliable estimates of the prevalence of dietary supplement use because of differences in definitions, varying frequency of use, and the increasing complexity of dietary supplement formulations [4]. National studies indicate many adults in the United States (US) use some form of CAM. For example, data from the National Health Interview Survey (NHIS) has consistently found approximately one third of US adults aged 18 and older reported using CAM in the previous 12 months (32.3% in 2002, 35.5% in 2007, and 33.2% in 2012) [5]. Another study found similar results, with 35% of US adults having used CAM in 2002, and a consistent trend of widespread use since 1997 [6]. However, a 2004 study reported that CAM use was as high as 48% among US adults [7].

As a result of their widespread use, $30.7 billion were spent on vitamins and nutritional supplements in the US in 2018 [8]. Furthermore, there is the potential for drug interactions with concomitant use of medications and dietary supplements, given that approximately one-quarter of individuals taking a prescription medication also take a dietary supplement [9–11]. Dietary supplements are not subjected to the rigorous premarketing approval process required for prescription drugs, thus knowledge of adverse effects and interactions between dietary supplements and medications is often poor.

An additional challenge to obtaining reliable knowledge of dietary supplement use is the lack of communication between physicians and patients on the use of CAM. [12]. However, patients may discuss dietary supplement use with their pharmacist, highlighting an important role for pharmacists in the safe use of dietary supplements [13]. Although dietary supplements are often available for sale in community pharmacies and pharmacists are considered an accessible resource for professional healthcare advice, it is not known whether pharmacists are knowledgeable about dietary supplements.

As a profession responsible for the appropriate use of medications, and thus identifying potential drug interactions with dietary supplements, this study sought to describe pharmacists’ personal use of vitamins, minerals, herbals and other dietary supplements, their reasons for use, the conditions used to treat, whether use is associated with demographic characteristics, and to whom they would recommend these products.

## Methods

### Study design and population

This cross-sectional study was conducted using survey data from a sample of pharmacists licensed in the state of Arizona, United States. Any pharmacist who had an email address on file with the state board of pharmacy in May 2015 was eligible for inclusion in this study. The primary purpose of the study was to identify the types, reasons, for using vitamins, minerals, herbals and other dietary supplements, and their recommendations to others. Therefore, a sample based on willingness to respond was acceptable. The University Institutional Review Board approved this study.

### Survey development

The questionnaire was developed using items derived from the 2007 National Health Interview Survey (NHIS) [14]. The questionnaire was designed using Qualtrics survey software (Qualtrics Labs, Inc. 2013) and was adaptive in nature, with the ability to display subsequent questions according to the pharmacists’ response to the previous question. For example, if the pharmacist indicated that they had ever used a particular dietary supplement, then the following question would ask about the reasons they used that particular product before moving on to the next product. Pharmacists were able to select multiple reasons for use for each product. The questionnaire included 103 items divided into four sections. The first section asked pharmacists to indicate their use of 10 different vitamin and mineral products. If pharmacists reported that they used a particular product, they were then asked to indicate: the reason(s) for use, the condition(s) they were attempting to treat, and if they would recommend the product to others (patients, family, or friends). The second section asked pharmacists to indicate their use of 21 herbal and other dietary supplements that included several herbs as well as chondroitin, Coenzyme Q10, creatine, fish oil, glucosamine, probiotics and melatonin (i.e., dietary supplements that are not considered vitamins or minerals). Similar to the first section, appropriate follow-up questions were posed if pharmacists indicated their use for the product. The third section posed two questions about pharmacists’ perceived safety and effectiveness of dietary supplements, and reasons why individuals never used any of these products. In the final section, four demographic questions were asked, including: age, gender, type of practice setting (community, institutional, academic and others), and location of practice setting (rural or non-rural). A community pharmacy research specialist at The University of Arizona reviewed the survey items for structure and consistency with the study objectives.

### Data collection

An introductory email describing the purpose of the study was sent to all licensed pharmacists in the State of Arizona using a list serve provided by the Arizona State Board of Pharmacy. One week later another email containing a link to the Qualtrics survey was sent to the same individuals. Instructions were provided within the questionnaire, and all pharmacists were advised that their participation was voluntary and that their responses would be anonymous. A reminder email was sent 2 weeks after the survey email, and the survey closed a further 2 weeks later.

### Statistical analysis

Data on vitamins, minerals, herbals and other dietary supplements were summarized using frequency counts and percentages. Differences in demographic characteristics between those who used vitamins, minerals, herbals and other dietary supplements, and those who did not, were compared using chi-square tests. A Pearson chi-square test was conducted to determine if there was any association between pharmacists’ use of vitamins, minerals, herbals and other dietary supplements and to whom they were recommended. An alpha level of 0.05 was selected a priori. All analyses were conducted using the SPSS computer software package (IBM Corp., Version 21.0. Armonk, NY).

## Results

### Demographic characteristics of study pharmacists

The demographic characteristics of the study pharmacists are shown in Table 1 stratified by use of vitamins and minerals (the vitamin/mineral group), and herbals and other dietary supplements (the herbal/other group). A total of 639 pharmacists completed the survey.

**Table 1:** Demographic characteristics of study pharmacists who used versus never used vitamins, minerals, herbals, and other dietary supplements

Characteristic	Used Vitamins/Minerals N (%)	Never Used Vitamins /Minerals N (%)	*p* value	Used Herbals/other dietary supplements N (%)	Never Used Herbals/other dietary supplements N (%)	*p* value
Gender	*n* = 498	*n* = 26	0.031	*n* = 268	*n* = 260	0.039
Male	193 (39	16 (62)		96 (36)	116 (45)	
Female	305 (61)	10 (38)		172 (64)	144 (55)	
Age, years	*n* = 490	n = 26	0.943	*n* = 263	*n* = 258	< 0.001
< 25	11 (2)	1 (4)		6 (2)	6 (2)	
25–34	125 (26)	5 (19)		50 (19)	80 (31)	
35–44	109 (22)	6 (23)		53 (20)	62 (24)	
≥45	245 (50)	14 (54)		154 (59)	110 (43)	
Type of practice setting	*n* = 492	n = 26	0.934	n = 268	n = 258	0.521
Community	207 (42)	10 (38)		115 (43)	106 (41)	
Institutional	139 (28)	7 (27)		71 (26)	76 (29)	
Academic	24 (5)	1 (4)		10 (4)	15 (6)	
Other	122 (25)	8 (31)		72 (27)	61 (24)	
Location of practice setting	*n* = 495	n = 26	0.938	*n* = 267	*n* = 260	0.660
Rural	56 (11)	3 (12)		32 (12)	28 (11)	
Non-rural	439 (89)	23 (88)		235 (88)	232 (89)	

A total of 639 respondents completed the survey, however they were not required to answer all questions thus the numbers in Table 1 do not always equal the total sample size. *p* values indicate differences between those who have used and those who have never used vitamins/minerals, and those who have used and those who have never used herbals/other dietary supplements

Use of vitamins/minerals was associated with gender, with more females using vitamins/minerals than males (61% versus 39%, *p* = 0.031). Use of vitamins/minerals was not associated with age, type of practice setting, or location of practice setting.

Use of herbals/other dietary supplements was associated with both gender and age, with more females using herbals/other dietary supplements than males (64% versus 36%, *p* = 0.039) and large differences between the youngest and oldest groups (*p* < 0.001). Use of herbals/other dietary supplements was not associated with type of practice setting or location of practice setting.

**Fig. 1:**
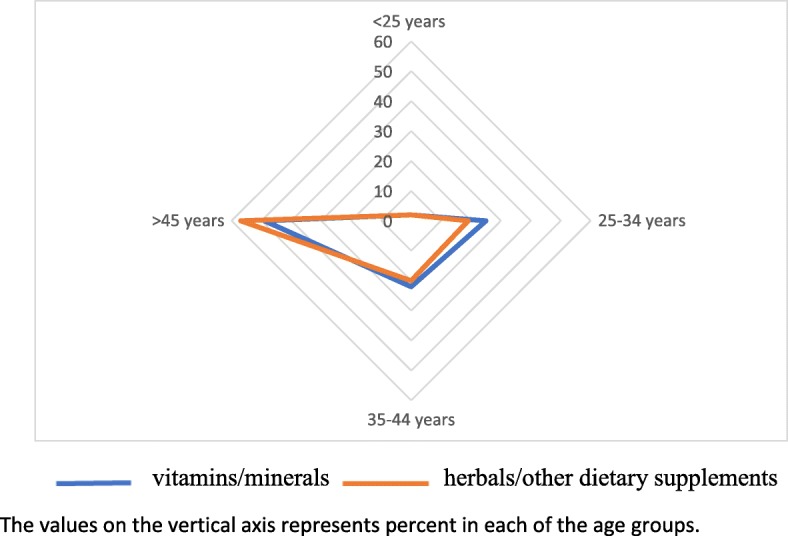
Radar chart indicating vitamin/mineral and herbals/other dietary supplements use by age groups

**Fig. 2:**
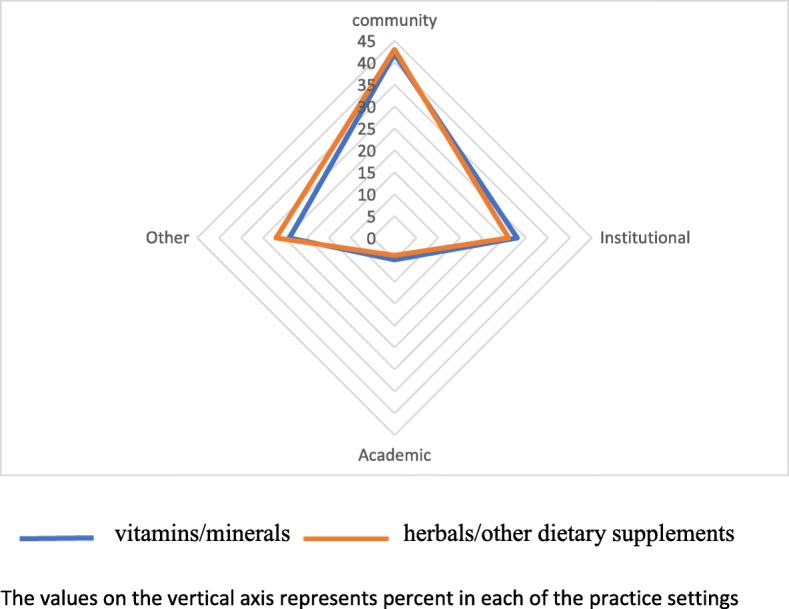
Radar chart indicating vitamin/mineral and herbals/other dietary supplements use by practice settings

The radar diagrams provide a graphical representation that the pattern of use of vitamins/minerals and herbals/other dietary supplements use is similar regardless of age (Fig. 1) or location of practice sites (Fig. 2).

### Perceived safety and effectiveness

A total of 528 pharmacists responded to the questions about safety and effectiveness; 312 (59%) reported that they thought dietary supplements were safe but only 169 (32%) reported they were effective.

### Use of vitamins and minerals

The most commonly used vitamin/mineral products in this sample of pharmacists were multivitamins (*n* = 579, 91%) and vitamin C (*n* = 453, 71%), while the least commonly used were chromium (*n* = 154, 24%) and magnesium (*n* = 223, 35%). The most commonly reported reason for use of different vitamin and mineral products in our survey was for general health and wellness (ranging from 47 to 90% of respondents for different vitamins and minerals). See Table 2 for further details.

**Table 2:** Pharmacists’ self-reported reasons for using vitamins/minerals

Item	Ever used N (%)	General health and wellness N (%)	Treat a specific disease N (%)	Improve physical health N (%)	Improve mental ability or memory N (%)	Suggested by healthcare professional N (%)	Suggested by family, friend or co-worker N (%)	Others N (%)
Vitamins/minerals								
Multivitamin	579 (91)	523 (90)	40 (7)	94 (15)	46 (8)	74 (13)	14 (2)	84 (11)
B Vitamin	402 (63)	303 (75)	62 (15)	92 (23)	52 (13)	55 (14)	17 (4)	33 (8)
Vitamin C	453 (71)	336 (74)	118 (26)	30 (6)	5 (1)	26 (6)	18 (4)	42 (9)
Vitamin D	434 (68)	265 (61)	114 (26)	25 (6)	18 (4)	148 (34)	10 (2)	31 (7)
Calcium	430 (67)	302 (70)	98 (23)	54 (12)	0	104 (24)	7 (1)	40 (9)
Chromium	154 (24)	92 (60)	38 (25)	35 (18)	2 (1)	13 (8)	5 (1)	44 (29)
Folic acid	300 (47)	182 (61)	59 (20)	22 (7)	11 (4)	90 (30)	4 (1)	60 (20)
Iron	274 (43)	129 (47)	117 (43)	21 (6)	2 (1)	81 (30)	3 (1)	35 (12)
Magnesium	223 (35)	135 (61)	72 (32)	32 (15)	5 (2)	41 (18)	4 (1)	37 (16)
Zinc	258 (40)	146 (57)	104 (40)	21 (8)	4 (2)	29 (11)	12 (5)	43 (14)

Others included: Prescription medications/medical treatment was too expensive, or medical treatment did not help

### Use of herbals and other dietary supplements

The most commonly used herbal/other dietary supplements in this sample of pharmacists were fish oil (*n* = 322, 65%), probiotics (*n* = 233, 53%), and fiber (*n* = 230, 53%), while the least commonly used were ephedra (*n* = 29, 5%), cinnamon (*n* = 53, 8%), and creatine (*n* = 60, 9%). The most commonly reported reason for use of herbals and other dietary supplements were for general health and wellness (range 17 to 81%) or to treat a specific disease (range 9 to 76%). See Table 3 for further details.

**Table 3:** Pharmacists’ self-reported reasons for using herbal and other dietary supplements

Item	Ever used N (%)	General health and wellness N (%)	Treat a specific disease N (%)	Improve physical health N (%)	Improve mental ability or memory N (%)	Suggested by healthcare professional N (%)	Suggested by family, friend or co-worker N (%)	Others N (%)
Cinnamon	53 (8)	26 (49)	32 (60)	3 (5)	2 (2)	2 (2)	3 (3)	15 (28)
Chondroitin	135 (21)	42 (31)	81 (60)	42 (30)	0	19 (14)	8 (6)	16 (12)
Coenzyme Q10	123 (19)	87 (71)	24 (20)	23 (18)	10 (8)	25 (20)	3 (2)	17 (14)
Cranberry	70 (11)	18 (26)	53 (76)	2 (3)	0	8 (11)	1 (1)	21 (30)
Creatine	60 (9)	13 (22)	0	65 (58)	0	1 (2)	3 (5)	12 (20)
Echinacea	141 (22)	49 (35)	91 (65)	0	1 (1)	7 (5)	15 (11)	22 (16)
Ephedra	29 (5)	5 (17)	8 (28)	10 (27)	5 (17)	1 (3)	1 (3)	14 (48)
Fiber/Psyllium	230 (53)	96 (42)	136 (59)	3 (1)	0	32 (14)	2 (1)	20 (9)
FishOil/Omega-3	322 (65)	227 (71)	87 (27)	23 (5)	31 (10)	57 (18)	9 (3)	34 (7)
Flaxseed Oil	111 (35)	90 (81)	22 (20)	9 (5)	8 (7)	15 (14)	3 (3)	20 (17)
Garlic	44 (25)	30 (68)	18 (41)	3 (7)	1 (2)	1 (2)	2 (5)	17 (39)
Ginseng	54 (26)	31 (57)	5 (9)	15 (24)	20 (37)	0	2 (4)	18 (24)
Glucosamine	150 (40)	50 (33)	85 (57)	60 (27)	0	11 (7)	7 (5)	11 (7)
Green Coffee Bean Extract	49 (25)	22 (45)	12 (24)	10 (20)	3 (6)	2 (4)	3 (5)	14 (29)
Green Tea	54 (26)	31 (57)	10 (19)	19 (28)	10 (19)	2 (4)	3 (6)	15 (28)
Melatonin	180 (45)	0	119 (66)	4 (2)	5 (3)	18 (10)	11 (6)	46 (26)
Probiotic	233 (53)	153 (66)	103 (44)	7 (3)	2 (1)	0	26 (11)	21 (11)
Saw Palmetto	36 (23)	17 (47)	18 (50)	0	0	2 (6)	5 (14)	8 (22)
Senna	98 (33)	21 (21)	69 (70)	0	0	0	15 (15)	18 (17)
Soy Isoflavones	22 (21)	13 (59)	11 (50)	3 (14)	2 (9)	0	1 (5)	11 (41)
St. John’s Wort	23 (22)	7 (30)	12 (52)	0	6 (26)	1 (4)	4 (17)	7 (30)

Others included: Prescription medications/medical treatment was too expensive, or medical treatment did not help

### Treatment of health conditions

Pharmacists were also asked which vitamins, minerals, herbals, or other dietary supplements they used to treat a variety of health conditions. Commonly used vitamins and minerals for particular conditions included: vitamin D for vitamin deficiencies (*n* = 140) and vitamin C for common cold (*n* = 111). Commonly used herbals and other dietary supplements for particular conditions included: melatonin for insomnia (*n* = 120) and fiber/psyllium and probiotics for stomach or intestinal illness (*n* = 114 and *n* = 103 respectively). See Additional file 1: Table S1 for further details.

The most commonly reported reasons for not using dietary supplements were: medical science has not shown that it works (5.6%), don’t need it (5.5%); and don’t believe it works (4.7%).

### Recommending vitamin, mineral, herbal, and other dietary supplements

**Fig. 3:**
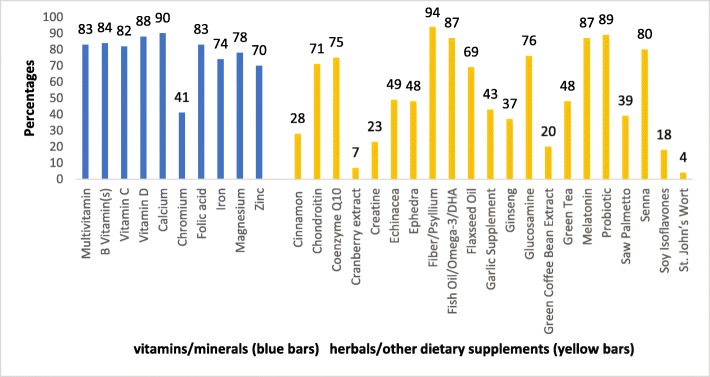
Percent of pharmacists recommending vitamin/mineral and herbals/other dietary supplements to patients, family and friends

Pharmacists in this survey indicated that they would recommend many of the dietary supplements to patients, friends, and family. The percent of endorsement ranged from a low of 4% for St. John’s Wort to a high of 94% for fiber/psyllium. For further details, see Fig. 3. There was no significant difference in the proportions of pharmacists who would recommend the use of multivitamins to patients, family and friends between pharmacists who used multivitamins and those who did not (*p* = 0.418).

## Discussion

In this study, we found that a substantial number of pharmacists used vitamins and minerals more than herbals and other dietary supplements, and that reasons for their use varied based on the type of products. We also observed distinct patterns in the selective use of vitamins, minerals, herbals, and other dietary supplements. To the best of our knowledge, this is the first survey conducted in the State of Arizona to investigate pharmacist’s use of dietary supplements, as well as their reasons for use, conditions they sought to treat, and their recommendations to patients, family, and friends. This is valuable information given that pharmacists are frontline healthcare professionals who may be asked to provide advice to patients about these products.

The demographic characteristics of our study pharmacists were similar to pharmacists in previously published studies, with more female pharmacists than males and a mean age of 46 ± 15 years [13, 15, 16]. A positive association was found between age group and use of herbals/other dietary supplements. This is not surprising given that adults aged 45 years and older tend to use more supplements for various reasons [17].

The high rate of vitamins/minerals (78%) and herbals/other dietary supplements (42%) used in our sample exceeds the rates reported in other studies of health professionals. For example, a survey of 533 pharmacists in Minnesota found 53% used dietary supplements, and that Echinacea (40%), aloe (37%) and zinc (36%) were the three most commonly used [18]. In comparison, the most commonly used dietary supplements in our survey were multivitamins (91%), vitamin C (71%), vitamin D (68%) and fish oil/omega-3 (65%). Axon et al. also found that fish oil and omega-3 were the most commonly used dietary supplements among student pharmacists [19]. Furthermore, a 2014 survey of dietary supplement use in the US conducted by the council for responsible nutrition reported that 75% of people used multivitamins, 20% used vitamin D, and 24% used calcium [20]. Likewise, Gardiner et al. and Kemper et al. found that multivitamins, calcium, B vitamins, vitamin C, and fish oil were among the most commonly used CAM products in their surveys targeting healthcare professionals, [16, 21] while multivitamins, calcium, and vitamin E were reported to be commonly used by the general population [22]. Additional research to investigate these variations is needed; The higher rates reported in our study compared to others may be because we specifically asked about numerous commonly used vitamins, minerals, herbals, and other dietary supplements or may be because we asked about ever use rather than a shorter timeframe. Our study findings support previous reports that pharmacists use these dietary supplements and provide new evidence that indicates use of these products has increased over the past decade. If this trend is also realized in the general population, then this finding has important clinical implications to ensure that pharmacists are able to identify any drug interactions and counsel patients on the appropriate use of dietary supplements.

Overall, pharmacists in our study recommended vitamins, minerals, and herbal products to patients, family, and friends in a similar pattern to their personal use of these products. However, their recommendations varied based on the type of product, for example the majority of pharmacists would recommend fiber/psyllium (94%) while very few would recommend St. John’s Wort (4%). This finding is similar to that of a 2001 survey of 420 pharmacists’ patterns of dietary supplement use [20].

Pharmacists in our study reported use of dietary supplements for both preventative and curative purposes. Previous studies have reported similar findings, where the main use of these products was to improve general health, to promote wellbeing, and to cure specific diseases [15, 23]. In our study, dietary supplements were most often used for chronic ailments and vitamin deficiencies, which concurs with the findings of other studies [24, 25]. However, we also observed that specific vitamins and minerals (such as vitamin C and zinc) and herbals and other dietary supplements (such as Echinacea, fiber/psyllium, and probiotics) were used to treat acute conditions such as cold, flu, diarrhea, and infections.

Over half (57%) of the pharmacists in our sample thought dietary supplements were safe, while approximately a third (32%) thought they were effective. These findings parallel those of previous US studies, which found that approximately 50% of pharmacists believed dietary supplements are safe [18, 26]. Meanwhile, 48% of pharmacists from California agreed that alternative medications are effective [26] yet only 19% of a smaller sample of pharmacists from Minnesota believed they are effective [18]. These results indicate variation in pharmacists’ perceptions of dietary supplements safety and effectiveness, which may be accounted for by differences in the definitions or types of products included in a specific survey. We recommend that pharmacists may benefit from improving their knowledge about the safety and efficacy of specific dietary supplements, so that they can better inform patients who use these products.

There were limitations associated with this study. Only 25% of respondents were male pharmacists and given that females generally have greater interest in complementary therapies, gender-specific self-selection issues might limit the generalizability of these findings. Data are also based on self-report from the pharmacist rather than direct observation or medical record review. Being a self-administered questionnaire, response bias is likely. The generalization of the results is limited as the sample of pharmacists was taken from one state alone, which may not be representative of all pharmacists in the State or in the US. Since pharmacists were asked about vitamins, minerals, and herbal and other dietary supplements used ever by them, there is chance of recall bias, as they may find it difficult to remember exactly which products were used. We used a previously validated questionnaire developed by NHIS, although we acknowledge the validity of the survey questions was not tested for our sample of respondents.

Future research in this area is warranted and should focus on assessing the patterns of dietary supplement use among pharmacists in other states and reasons for use among different healthcare professionals. This research should then be compared to use and reasons for use in the general population.

## Conclusion

Our study found that pharmacists were selective in their use of dietary supplements, and that the types of supplements they used were similar to those in previously published studies. However, a new important finding from our study was that these products were used at higher rates than previously reported, which suggests use of dietary supplements is increasing and may have clinical implications for the identification of potential interactions and counseling on the appropriate use of these products. Our study also identified pharmacists’ reasons for use, which concurred with previously published studies. Pharmacists in our study recommended some of the more commonly used products to patients, family and friends. This is valuable information given that pharmacists are frontline healthcare professionals who may be asked to provide advice to patients about these products. Future research should investigate some of the discrepancies between our findings and those of previous studies, as well as investigating dietary supplement use in broader populations.

## Additional file

Additional file 1: **Table S1.** Pharmacists’ self-reported conditions for using vitamins/minerals, and herbal/other dietary supplements were used to treat. (DOCX 17 kb)

## Data Availability

The datasets used and/or analyzed during the current study are available from the corresponding author on reasonable request.

## Abbreviations

CAM: complementary and alternative medicine
NHIS: National Health Interview Survey
NIH: National Institute of Health
SD: Standard deviation
US: United States
