# Supplementary material for: How do pharmacists use and recommend vitamins, minerals, herbals and other dietary supplements?
Source: BMC Complement Altern Med. 2019 Aug 22;19:229. doi: 10.1186/s12906-019-2637-y (PMC6704661; doi:10.1186/s12906-019-2637-y)
Supplement: Supplementary file 1 — Table S1. Pharmacists’ self-reported conditions for using vitamins/minerals, and herbal/other dietary supplements were used to treat. (DOCX 17 kb) [file 12906_2019_2637_MOESM1_ESM.docx]

**Supplementary File**

Supplementary Table 1: Pharmacists’ self-reported conditions for using vitamins/minerals, and herbal/other dietary supplements were used to treat

| Item, N responded | Anxiety or Depression  N (%) | Arthritis or Joint pain/Stiffness  N (%) | Common  cold  N (%) | Heart Health  N (%) | Insomnia  N (%) | Stomach or intestinal illness  N (%) | Vitamin  Deficiency  N (%) | Others  N (%) |
| --- | --- | --- | --- | --- | --- | --- | --- | --- |
| Vitamins/minerals | | | | | | | | |
| Multivitamin, 579 | 6 (1) | 6 (1) | 5 (1) | 17 (3) | 7 (1) | 8 (1) | 33 (6) | 21 (11) |
| B Vitamin, 402 | 7 (2) | 4 (1) | 3 (1) | 6 (1) | 1 (<1) | 1 (<1) | 36 (9) | 67 (16) |
| Vitamin C, 453 | 0 | 6 (1) | 111 (25) | 7 (2) | 0 | 0 | 21 (5) | 49 (10) |
| Vitamin D, 434 | 8 (2) | 20 (5) | 2 (1) | 15 (4) | 2 (1) | 4 (1) | 140 (32) | 87 (18) |
| Calcium, 430 | 0 | 25 (6) | 0 | 10 (2) | 2 (1) | 17 (4) | 31 (5) | 125 (17) |
| Chromium, 154 | 0 | 0 | 5 (3) | 1 (1) | 1 (1) | 0 | 3 (2) | 45 (10) |
| Folic acid, 300 | 3 (10) | 2 (1) | 0 | 12 (4) | 0 | 0 | 20 (7) | 111 (36) |
| Iron, 274 | 0 | 2 (1) | 0 | 3 (1) | 1 (<1) | 1 (<1) | 76 (28) | 96 (34) |
| Magnesium, 223 | 4 (2) | 8 (4) | 0 | 19 (9) | 11 (5) | 11 (5) | 14 (6) | 76 (22) |
| Zinc, 258 | 0 | 3 (1) | 81 (31) | 3 (1) | 2 (1) | 0 | 10 (4) | 44 (16) |
| Herbal/other dietary supplements | | | | | | | | |
| Cinnamon, 53 | 0 | 0 | 0 | 0 | 0 | 0 | 34 (64) | 1 (2) |
| Chondroitin, 135 | 0 | 82 (61) | 13 (10) | 0 | 0 | 0 | 0 | 6 (4) |
| Coenzyme Q10, 123 | 0 | 3 (2) | 1 (1) | 0 | 21 (17) | 1 (1) | 1 (1) | 21 (17) |
| Cranberry, 70 | 0 | 1 (1) | 1 (1) | 0 | 0 | 0 | 0 | 57 (81) |
| Creatine, 60 | 0 | 0 | 1 (2) | 0 | 0 | 0 | 0 | 2 (3) |
| Echinacea, 141 | 0 | 0 | 0 | 88 (62) | 0 | 0 | 1 (1) | 7 (5) |
| Ephedra, 29 | 0 | 0 | 0 | 6 (20) | 0 | 0 | 1 (3) | 4 (14) |
| Fiber/Psyllium, 230 | 0 | 0 | 0 | 0 | 6 (3) | 114 (50) | 1 (<1) | 41 (18) |
| FishOil/Omega-3, 322 | 3 (1) | 19 (6) | 4 (1) | 74(23) | 0 | 1 (<1) | 3 (1) | 59 (18) |
| Flaxseed Oil, 111 | 1 (1) | 3 (2) | 0 | 0 | 18 (16) | 0 | 0 | 19 (15) |
| Garlic, 44 | 0 | 1 (2) | 0 | 8 (18) | 7 (16) | 0 | 1 (2) | 11 (19) |
| Ginseng, 54 | 0 | 1 (2) | 0 | 0 | 0 | 0 | 1 (2) | 2 (4) |
| Glucosamine, 150 | 0 | 85 (57) | 14 (9) | 0 | 0 | 0 | 2 (1) | 6 (4) |
| Green Coffee Bean Extract, 49 | 0 | 0 | 0 | 0 | 0 | 0 | 3 (6) | 11 (22) |
| Green Tea, 54 | 0 | 0 | 0 | 0 | 1 (2) | 2 (4) | 0 | 14 (20) |
| Melatonin, 180 | 1 (1) | 0 | 0 | 0 | 120 (67) | 1 (1) | 0 | 8 (4) |
| Probiotic, 233 | 1 (<1) | 2 (1) | 1 (<1) | 0 | 0 | 103 (44) | 0 | 23 (10) |
| Saw Palmetto, 36 | 0 | 0 | 0 | 0 | 0 | 0 | 0 | 21 (58) |
| Senna, 98 | 0 | 0 | 0 | 0 | 0 | 54 (55) | 0 | 23 (24) |
| Soy Isoflavones, 22 | 1 (5) | 0 | 0 | 0 | 0 | 0 | 0 | 14 (55) |
| St. John’s Wort, 23 | 14 (61) | 0 | 0 | 0 | 0 | 0 | 0 | 0 |
